# Evaluation of the interest to combine a CD4 Th1-inducer cancer vaccine derived from telomerase and atezolizumab plus bevacizumab in unresectable hepatocellular carcinoma: a randomized non-comparative phase II study (TERTIO – PRODIGE 82)

**DOI:** 10.1186/s12885-023-11065-0

**Published:** 2023-07-29

**Authors:** Angélique Vienot, Marion Jacquin, Magali Rebucci-Peixoto, Dimitri Pureur, François Ghiringhelli, Eric Assenat, Pascal Hammel, Olivier Rosmorduc, Morgane Stouvenot, Manon Allaire, Mohamed Bouattour, Hélène Regnault, Serge Fratte, Eric Raymond, Emilie Soularue, Stéphanie Husson-Wetzel, Vincent Di Martino, Allison Muller, Anne-Laure Clairet, Christine Fagnoni-Legat, Olivier Adotevi, Aurélia Meurisse, Dewi Vernerey, Christophe Borg

**Affiliations:** 1 Department of Medical Oncology, University Hospital of Besançon, 25000 Besançon, France; 2 Clinical Investigational Center, CIC-1431 Besançon, France; 3 INSERM, EFS BFC, UMR1098, RIGHT, University of Bourgogne Franche-Comté, Interactions Greffon-Hôte-Tumeur/Ingénierie Cellulaire Et Génique, Besançon, France; 4 Groupe Coopérateur Multidisciplinaire en Oncologie (GERCOR) Oncology Multidisciplinary Group, Paris, France; 5 Department of Hepatology, University Hospital of Besançon, Besançon, France; 6 Department of Medical Oncology, Georges François Leclerc Cancer Center-UNICANCER, Dijon, France; 7 Department of Medical Oncology, Saint Eloi Hospital, University Hospital, Montpellier of Montpellier, France; 8 Department of Digestive and Medical Oncology, Paul-Brousse Hospital, Villejuif, France; 9 Department of Hepato-Biliary, Paul-Brousse Hospital, Villejuif, France; 10 Department of Gastroenterology, University Hospital of Besançon, Besançon, France; 11 Department of Hepatogastroenterology, Pitié Salpêtrière Hospital, Paris, France; 12 Department of Liver Cancer, Beaujon Hospital, Clichy, France; 13 Department of Gastroenterology and Hepatology, Henri Mondor Hospital, Creteil, France; 14 Department of Gastroenterology, Nord Franche Comté Hospital, Montbéliard, France; 15 Department of Medical Oncology, Paris Saint-Joseph Hospital, Paris, France; 16 Department of Medical Oncology, Institute Mutualiste Montsouris, Paris, France; 17 Department of Gastroenterology, Groupe Hospitalier de La Région Mulhouse Sud Alsace, Mulhouse, France; 18 Department of Clinical Research and Innovation, Vigilance Unit, University Hospital of Besançon, Besançon, France; 19 Department of Pharmacy, University Hospital of Besançon, Besançon, France; 20 Methodology and Quality of Life in Oncology Unit, University Hospital of Besançon, Besançon, France

**Keywords:** Hepatocellular carcinoma, Immunotherapy, Vaccination, Telomerase

## Abstract

**Background:**

Several cancer immunotherapies that target the PD-L1/PD-1 pathway show promising clinical activity in patients with hepatocellular carcinoma (HCC). However, the standard of care in first-line treatment with atezolizumab (anti-PD-L1 therapy) in combination with bevacizumab is associated with a limited objective response rate. Telomerase reverse transcriptase (TERT) activation meets the criteria of oncogenic addiction in HCC and could be actionable therapeutic target and a relevant tumor antigen. Therefore we hypothesized that combining anti-PD-1/PD-L1 therapy with an anti-telomerase vaccine might be an attractive therapy in HCC. UCPVax is a therapeutic cancer vaccine composed of two separate peptides derived from telomerase (human TERT). UCPVax has been evaluated in a multicenter phase I/II study in non–small cell lung cancers and has demonstrated to be safe and immunogenic, and is under evaluation in combination with atezolizumab in a phase II clinical trial in tumors where telomerase reactivation contributes to an oncogene addiction (HPV^+^ cancers). The aim of the TERTIO study is to determine the clinical interest and immunological efficacy of a treatment combining the CD4 helper T-inducer cancer anti-telomerase vaccine (UCPVax) with atezolizumab and bevacizumab in unresectable HCC in a multicenter randomized phase II study.

**Methods:**

Patients with locally advanced, metastatic or unresectable HCC who have not previously received systemic anti-cancer treatment are eligible. The primary end point is the objective response rate at 6 months. Patients will be allocated to a treatment arm with a randomization 2:1. In both arms, patients will receive atezolizumab at fixed dose of 1200 mg IV infusion and bevacizumab at fixed dose of 15 mg/kg IV infusion, every 3 weeks, according to the standard of care. In the experimental arm, these treatments will be combined with the UCPVax vaccine at 0.5 mg subcutaneously.

**Discussion:**

Combining anti-PD-1/PD-L1 therapy with an anti-telomerase vaccine gains serious consideration in HCC, in order to extend the clinical efficacy of anti-PD-1/PD-L1. Indeed, anti-cancer vaccines can induce tumor-specific T cell expansion and activation and therefore restore the cancer-immunity cycle in patients lacking pre-existing anti-tumor responses. Thus, there is a strong rational to combine immune checkpoint blockade therapy and anticancer vaccine (UCPVax) in order to activate antitumor T cell immunity and bypass the immunosuppression in the tumor microenvironment in HCC. This pivotal proof of concept study will evaluate the efficacy and safety of the combination of a CD4 Th1-inducer cancer vaccine derived from telomerase (UCPVax) and atezolizumab plus bevacizumab in unresectable HCC, as well as confirming their synergic mechanism, and settling the basis for a new combination for future clinical trials.

**Trial registration:**

NCT05528952.

## Background

Telomerase reactivation is observed in 90% of hepatocellular carcinoma (HCC) and seems to be a major step in tumor initiation [1, 2]. Up to 60% of cases are related to mutations in telomerase reverse transcriptase (TERT) promoter [3]. TERT is the main enzyme for the activity of telomerase and is classically expressed during embryogenesis in most cells including fetal hepatocytes, but is suppressed in most cells in the adult body. Telomerase reactivation is a key event in human carcinogenesis to avoid telomere shortening, senescence, and apoptosis and enables tumor cells to perform unrestrained rounds of proliferation. TERT activation meets the criteria of oncogenic addiction in HCC and could be actionable therapeutic target and a relevant tumor antigen [2].

Several cancer immunotherapies that target the PD-L1/PD-1 pathway (*i.e.*, checkpoint inhibitors) show promising clinical activity in patients with HCC. In particular, atezolizumab selectively targets PD-L1 to prevent interaction with receptors PD-1 and B7-1, thus reversing T-cell suppression. Moreover, atezolizumab in combination with bevacizumab, a monoclonal antibody that targets VEGF and inhibits angiogenesis, is associated with an objective response rate of 30% [4, 5]. This tumor response has led to FDA (Food and Drug Administration) and EMA (European Medicines Agency) approvals, in first-line treatment in unresectable HCC. Combinations studies evaluating anti-CTLA4 or tyrosine kinase inhibitor and anti-PD-1/PD-L1 antibodies displayed similar level of efficacy but at the price of increased toxicity in patients with comorbidities such as liver failure [6–10]. Therefore, improving the number of eligible patients for combination immunotherapies in this frailty population is currently an unresolved issue.

The success of most immunotherapies relies on CD8^+^ T cells effectively infiltrating tumors. Tumor-reactive CD4^+^ T cells have been found to ensure efficient effector CD8^+^ T cells recruitment at the tumor site [11–15]. Promoting tumor-specific Th1 CD4 activation might be an attractive therapeutic option to enhance anti-PD-1/PD-L1 efficacy. However, no option is currently available to expand tumor-specific Th1 lymphocytes in most patients. Then, we have used an optimized reverse immunology approach to identify four novel MHC class II-restricted peptides derived from human TERT referred as “Universal Cancer Peptides” (UCP) [16]. UCPVax is a therapeutic cancer vaccine developed by our team and composed of two separate peptides called UCP2 and UCP4 derived from telomerase. This vaccine, evaluated in two early clinical trials (NCT02818426 [17], NCT04280848), displays a good safety profile and improves anti-TERT CD4 Th1 in peripheral blood.

We have elaborated a collaborative network to investigate the clinical impact of this Th1-inducer vaccine with atezolizumab in tumors where telomerase reactivation contributes to an oncogene addiction: human papilloma virus (HPV)-induced cancers and HCC. Our team has already promoted a phase II clinical trial (VolATIL study, NCT03946358 [18]) in HPV^+^ cancers. This study combining the anti-telomerase vaccine with atezolizumab shows a favorable safety profile and promising long-lasting response and HPV ctDNA complete disappearance in the interim analysis. This combination highlights also the presence of tumor antigen-specific CD8 and CD4 T cells in tumor-infiltrating lymphocytes after the first step of vaccination.

Combining anti-PD-1/PD-L1 therapy with an anti-telomerase vaccine gains serious consideration in HCC, in order to extend the clinical efficacy of anti-PD-1/PD-L1. Indeed, anti-cancer vaccines can induce tumor-specific T cells expansion and activation and therefore restore the cancer-immunity cycle in patients lacking pre-existing anti-tumor responses. Recent data from preclinical studies support this strategy. In conclusion, there is a strong rational to combine immune checkpoint blockade therapy and anticancer vaccine (UCPVax) in order to activate antitumor T cell immunity and bypass the immunosuppression in the tumor microenvironment in HCC.

So in the present TERTIO clinical trial, we propose to determine the clinical interest and immunological efficacy of a treatment combining the CD4 helper T-inducer cancer anti-telomerase vaccine (UCPVax) with anti-PD-L1 therapy (atezolizumab) and bevacizumab in unresectable HCC by evaluation of the objective response rate at 6 months (randomized non-comparative phase II, 105 patients) according to RECIST v1.1 criteria. We will also decipher as a secondary objective, the molecular and immunological parameters determining the clinical outcomes.

## Methods and analysis

TERTIO study is a multicentre, non-comparative, randomized, open-label phase II trial, to evaluate a strategy combining UCPVax, atezolizumab and bevacizumab in advanced patients with unresectable HCC. This study was developed by the “National Institute of Health and Medical Research (INSERM), Unit 1098” and “Clinical Investigational Center (CIC) 1431”. The study is sponsored by University Hospital of Besançon and supported by INCa and DGOS (PHRC-K22-127), by the GERCOR collaborative group and A.R.C.A.D. Foundation. The data management, the methodology and the statistical analyses are undertaken by the “Methodology and Quality of Life Unit in Oncology” of the University Hospital of Besançon. The trial is registered on the clinicaltrials.gov database (NCT05528952).

The study has received the approval from Committee for Protection of Persons and by the French National Agency for the Safety of Medicines and Health Products on August 10, 2022 by Clinical Trials Information System (CTIS) portal, and will be conducted in accordance with the Declaration of Helsinki and the Good Clinical Practice.

### Study objectives

The primary objective of this clinical trial is to assess the efficacy of a strategy combining UCPVax and atezolizumab plus bevacizumab combination in patients with unresectable HCC by evaluation of the objective response rate at 6 months, according to RECIST v1.1.

*The secondary objectives* are:

1. Overall survival (OS)
2. Progression-free survival (PFS)
3. Disease control and objective response rates, according to RECIST v1.1 and imRECIST
4. Health-related quality of life (QoL)
5. Safety of UCPVax in association with anti-PD-L1 plus anti-angiogenic

*The ancillary analyses* are:

1. Correlation between the tumor genotyping for TERT promoter mutations, telomerase and PD-L1 expression and treatment efficacy
2. Evaluation of telomerase-specific T cell responses before and after treatment in peripheral blood mononuclear cells
3. Research of biomarkers

### Patient selection

The study population consists of patients with histologically confirmed HCC locally advanced, metastatic or unresectable. Patients should not have previously received systemic anti-cancer treatment.

Patients should have an Eastern Cooperative Oncology Group-performance status (ECOG-PS) of 0 or 1, a Child–Pugh Class A status and adequate organ function.

The main inclusion and exclusion criteria are listed in Table 1.

**Table 1:** Main inclusion and exclusion criteria of the TERTIO trial

**Inclusion criteria**
1. Age ≥ 18 years 2. Histologically confirmed hepatocellular carcinoma (HCC) 3. Locally advanced, metastatic, or unresectable disease 4. BCLC C stage or BCLC B stage not eligible to loco-regional therapy according to the Barcelona Clinic Liver Cancer (BCLC) staging system 5. Measurable disease defined according to RECIST v1.1 guidelines 6. Patient who had not previously received systemic anti-cancer treatment 7. Patients who have received previous chemoembolization, radioembolization and/or radiotherapy should have recovered from any treatment related toxicity, to a level of ≤ grade 1 (according to National Cancer Institute common terminology criteria for adverse events, version 5) with the exception of Grade 2 alopecia 8. ECOG-Performance status < 2 9. Child–Pugh Class A status 10. Documented virology status of hepatitis, as confirmed by screening HBV and HCV tests: - For patients with active HBV: HBV DNA < 500 IU/ml during screening, initiation of anti-HBV treatment at least 14 days prior to randomization and willingness to continue anti-HBV treatment during the study (per local standard of care) - Patients with HCV, either with resolved infection (as evidenced by detectable antibody) or chronic infection (as evidence by detectable HCV RNA), are eligible 11. Performance of an esophagogastroduodenoscopy and assessment and treatment of varices of all sizes per local standard of care prior to randomization
**Exclusion criteria**
1. Patients previously exposed to anti-tumor immunotherapy as anti-PD-1, anti-PD-L1, or anti-CTLA4 agent or any immune therapy 2. Diagnosis of additional malignancy within 3 years prior to the inclusion with the exception of curatively treated basal cell carcinoma of the skin and/or curatively resected in situ cervical or breast cancer 3. Know fibrolamellar HCC, sarcomatoid HCC, or mixed cholangiocarcinoma and HCC 4. Known active central nervous system metastases and/or carcinomatous meningitis. Subject with previously treated brain metastases and with radiological and clinical stability are allowed 5. History of encephalopathy 6. Prior bleeding event due to untreated or incompletely treated esophageal and/or gastric varices within 6 months prior to randomization 7. Uncontrolled pleural effusion, pericardial effusion, ascites or symptomatic fistula 8. Inadequate organ functions: known cardiac failure of unstable coronaropathy, respiratory failure, or uncontrolled infection or another life-risk condition 9. Patients with Left Ventricular Ejection Fraction (LEVF) < 40% 10. HIV positive (HIV 1/2 antibodies patients), or a known history of active Tuberculosis bacillus 11. Prior allogeneic bone marrow transplantation or prior solid organ transplantation 12. Active autoimmune disease that has required a systemic treatment in past 2 years (i.e. corticosteroids or immunosuppressive drugs) 13. Patients under chronic treatment with systemic corticoids or other immunosuppressive drugs (prednisone or prednisolone ≤ 10 mg/day is allowed) for a period of at least 4 weeks and whose treatment was not stopped 1 week prior to the start of the study treatment 14. Inadequate hematology function: Lymphocyte count at baseline < 800/mm^3^; neutrophil count < 1000/mm^3^, platelets < 75,000/mm^3^, Hemoglobin < 9 g/dL (patients may be transfused to meet this criterion) 15. Inadequate hepatic function: bilirubin threefold ULN, AST/ALT fivefold ULN, International normalized thromboplastin time ratio > 2 16. Inadequate renal function: MDRD CrCl < 30 ml/min, proteinuria ≥ 1 g/24h 17. Others inadequate laboratory values: serum albumin < 28 g/L; troponin > ULN; BNP > ULN

### Study description

#### Randomization

Once the patient’s consent is obtained, the Investigator will confirm that all required radiological and biological procedures were performed within schedule before randomization.

**Fig. 1:**
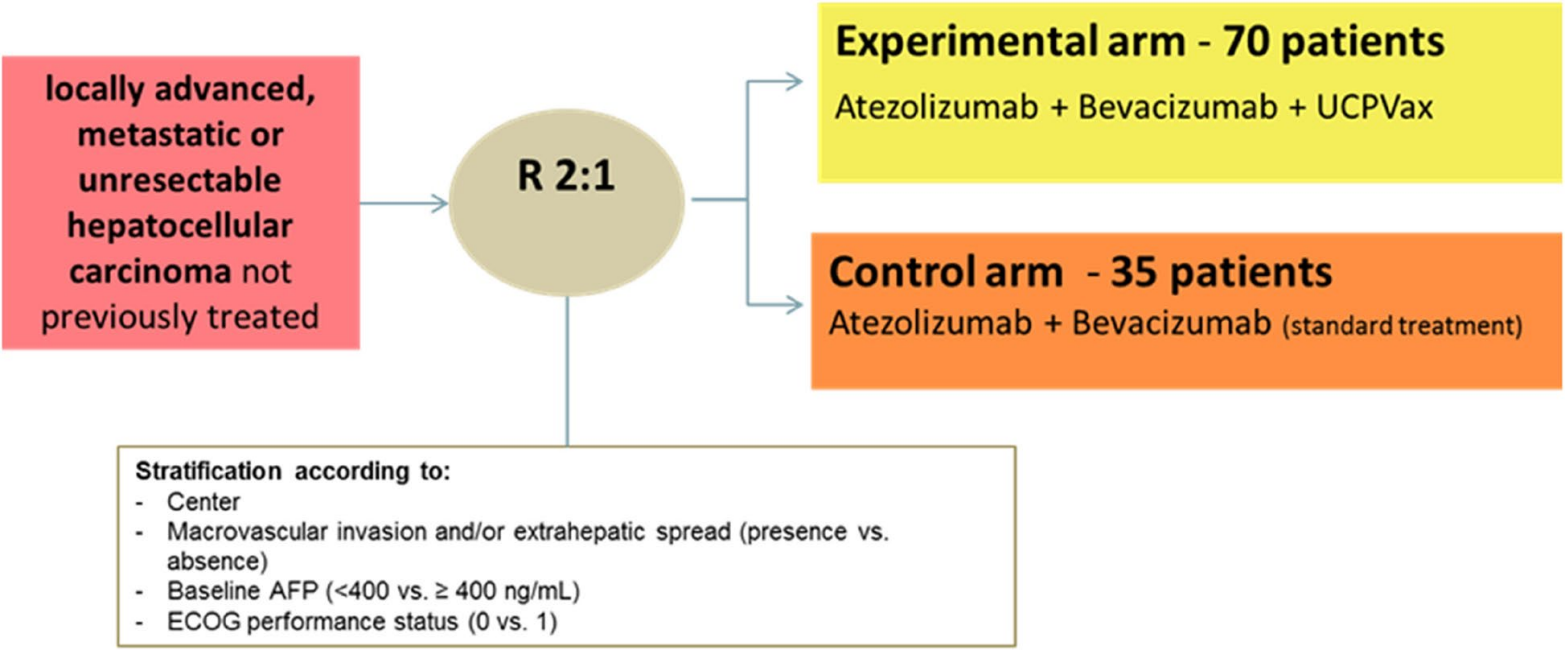
TERTIO study design

Patients will be randomized via the electronic case report form (eCRF) and allocated to a treatment arm (Arm A or B) with a randomization 2:1 (Fig. 1).

#### Therapeutic sequence

##### Experimental Arm (arm A): atezolizumab plus bevacizumab and UCPVax

UCPVax is a telomerase-derived cancer vaccine developed by academic research network at Besançon [17, 18]. The Good Manufacturing Practice (GMP) drug substance of UCPVax was produced by Provepharm life Solution (Marseille, France). Clinical grade peptide production and formulation will be performed according to GMP preparation and compliance by Pharmacy department of University Hospital of Besançon like in previous trial in lung cancer [17]. The dose of the vaccine used in the present trial will be 0.5 mg which guarantees a good immunological profil while limiting the adverse events related to Montanide.

The two UCPVax peptides: UCP2 and UCP4 at 0.5 mg/ml (combined with Montanide ISA51 as adjuvant), will be injected subcutaneously in two separate sites (one site per peptide) at days 1, 8, 15, 29, 36 and 43 (priming phase) following by boost vaccinations: every 6 weeks for the two first boosts (W13 and W19) and then every 9 weeks (W28, W37 and W46).

**Fig. 2:**
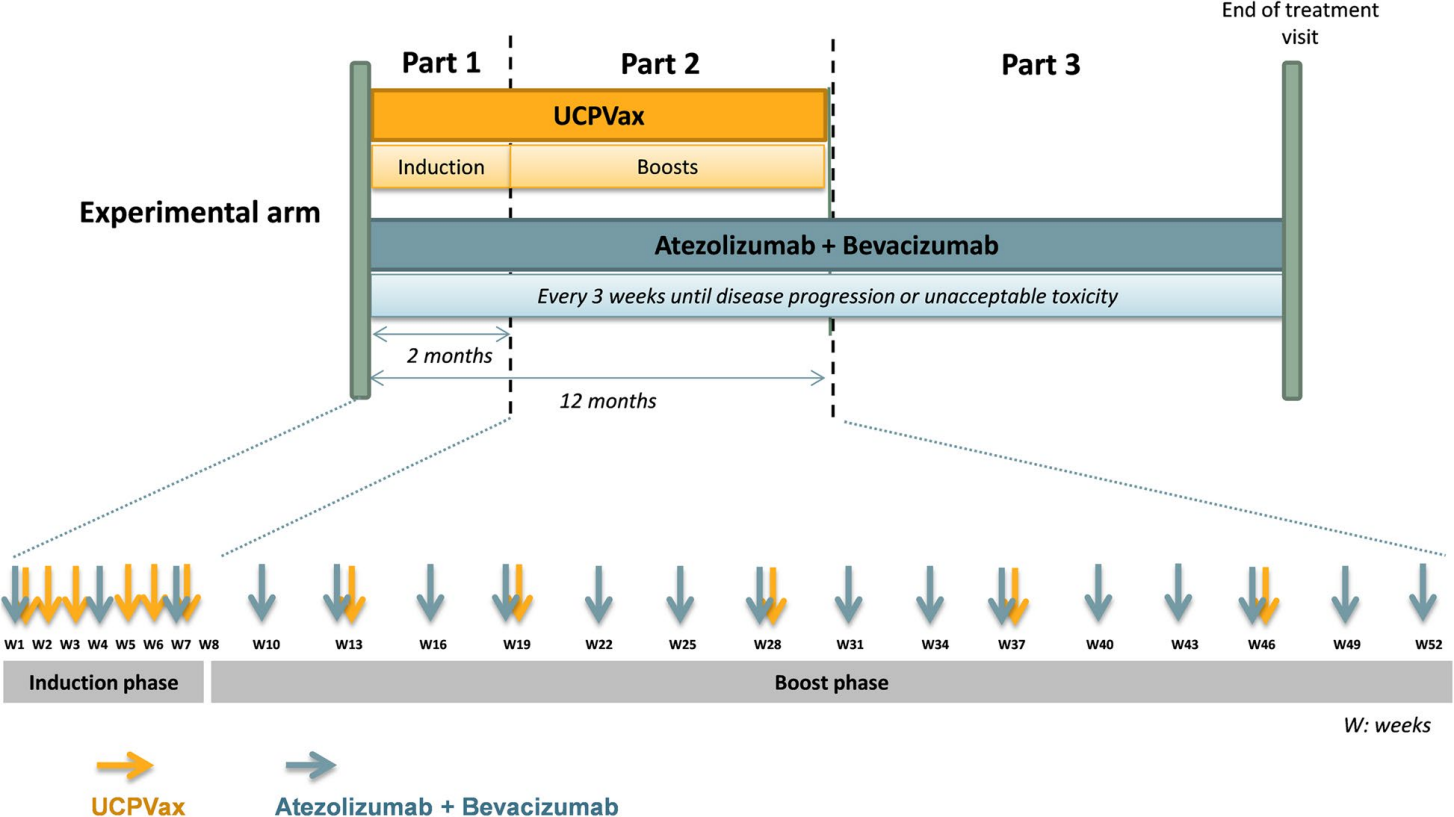
Treatment schedule in experimental arm

In combination, patients will receive atezolizumab at fixed dose of 1200 mg in 60 min IV infusion and bevacizumab at fixed dose of 15 mg/kg in 90 min IV infusion since Day 1, every 3 weeks, until disease progression or unacceptable toxicity or maximum 2 years in according to standard of care (Fig. 2). If atezolizumab is well tolerated at first cycle, atezolizumab perfusion can be reduced to 30 min in the following cycles. If bevacizumab is well tolerated at the first cycle, bevacizumab perfusion can be reduced to 60 min then 30 min in the following cycles.

When the three drugs will be administrated in the same visit, atezolizumab will be administered first followed by bevacizumab, and then by UCPVax with a minimum of 5 min between dosing.

##### Control Arm (arm B) – standard of care: atezolizumab plus bevacizumab

In arm B, patients will receive accordingly to standard of care: atezolizumab at fixed dose of 1200 mg IV infusion and bevacizumab at fixed dose of 15 mg/kg IV infusion since Day 1, every 3 weeks, until disease progression or unacceptable toxicity or maximum 2 years. Beyond the 2-year study period, and in the absence of disease progression or unacceptable toxicity, we recommend that investigators continue atezolizumab-bevacizumab treatment as part of standard practice.

#### Evaluation, Laboratory tests and follow-up

Tumor assessment will be carried out according to RECIST v1.1 criteria using total body CT scan performed every 8 weeks until progression, at end of vaccination visit, and at end of atezolizumab plus bevacizumab treatment visit. If progressive disease, it could be confirmed by another total body CT scan 4 to 8 weeks after the precedent. A liver MRI will be also planned at baseline, at W9, and at M6. Radiological assessments will be anonymized by the investigational centers and all imaging data will be collected at the end of the study for centralized review.

A health-related Qol EORTC-QLQ-C30 questionnaire will be collected at baseline, every 8 weeks until end of treatment visit, at end of vaccination visit, and at end of atezolizumab plus bevacizumab treatment visit.

Immunological safety assessment will be performed at baseline, W5 and W9 to analyse IgG, IgE, circulating antibodies (ANA, TPO), cytokine release and anti-drugs-antibodies.

#### Ancillary analyses

Ancillary studies will be conducted to determine the clinical interest and immunological efficacy of a treatment combining the CD4 helper T-inducer cancer anti-telomerase vaccine with anti-PDL1 therapy (atezolizumab) and bevacizumab in unresectable HCC, and to decipher the molecular parameters determining the clinical outcomes. Biomarkers correlated with combined immunotherapy efficacy will be determined.

Biomonitoring will be performed at baseline, at M2 and M6. A total of 50 ml of blood will be collected at each biomonitoring time point: six EDTA 6 ml tubes for PBMC collection, one EDTA 6 ml tube for plasma collection, and two EDTA 4 ml tube for plasma for ctDNA collection.

Tumor samples collection:

- Tumor samples obtained by biopsies or surgery at diagnosis will be centralized for translational research program, to measure by immunohistochemistry immune-related biomarkers, tumor genotyping and RNA sequencing analyses.
- A fresh tumor biopsy will be performed at progression and in case of surgery or radiofrequency after tumor response (optional). These tumor samples will be collected for translational research: RNA sequencing (RNA from the biopsy will be isolated and stored at -80 °C) and Antigen-specific T cell monitoring.

#### Data management

Data analysis will be similar to that from previous studies by our team [18–20]. For each patient enrolled in the TERTIO study, the investigators must document all required data in the corresponding source documents. These data must then be entered in eCRF, which will be accessible only by authorized persons via secured web connection. One eCRF will be created for each patient. The investigator has the responsibility for its completion, proof reading, as well as its approval after the final verification for the authenticity and accuracy of all entered data. The Monitor, who is mandated by the Sponsor, will ensure that the study is conducted in accordance with Good Clinical Practice guidelines and all applicable local laws and that the rights, security and well-being of the patient are respected. The Monitor will perform source document verification and validation and request clarification to ensure the accuracy, completeness, and reliability of data. The Investigator guarantees the Sponsor or its representative direct access to source documents. Throughout the study, data electronically captured via eCRF will be regularly checked for consistency, and queries on data clarification will be generated through eCRF. At the end of the data handling process, a data review meeting will be held to prepare the database lock. After database lock, data will be transferred into SAS format to produce statistical analyses.

### Statistical considerations

#### Determination of sample size

A total of 105 patients will be randomized using a 2:1 randomization ratio to allocate patients to either the experimental arm (Arm A: atezolizumab plus bevacizumab and UCPVax) or the control arm (Arm B: atezolizumab plus bevacizumab).

In the experimental Arm (Arm A), the aim is to demonstrate that the rate of patients with an objective response at 6 months post randomization (P) is clearly higher than 30% [5] (P0) which would not be satisfactory.

**Fig. 3:**
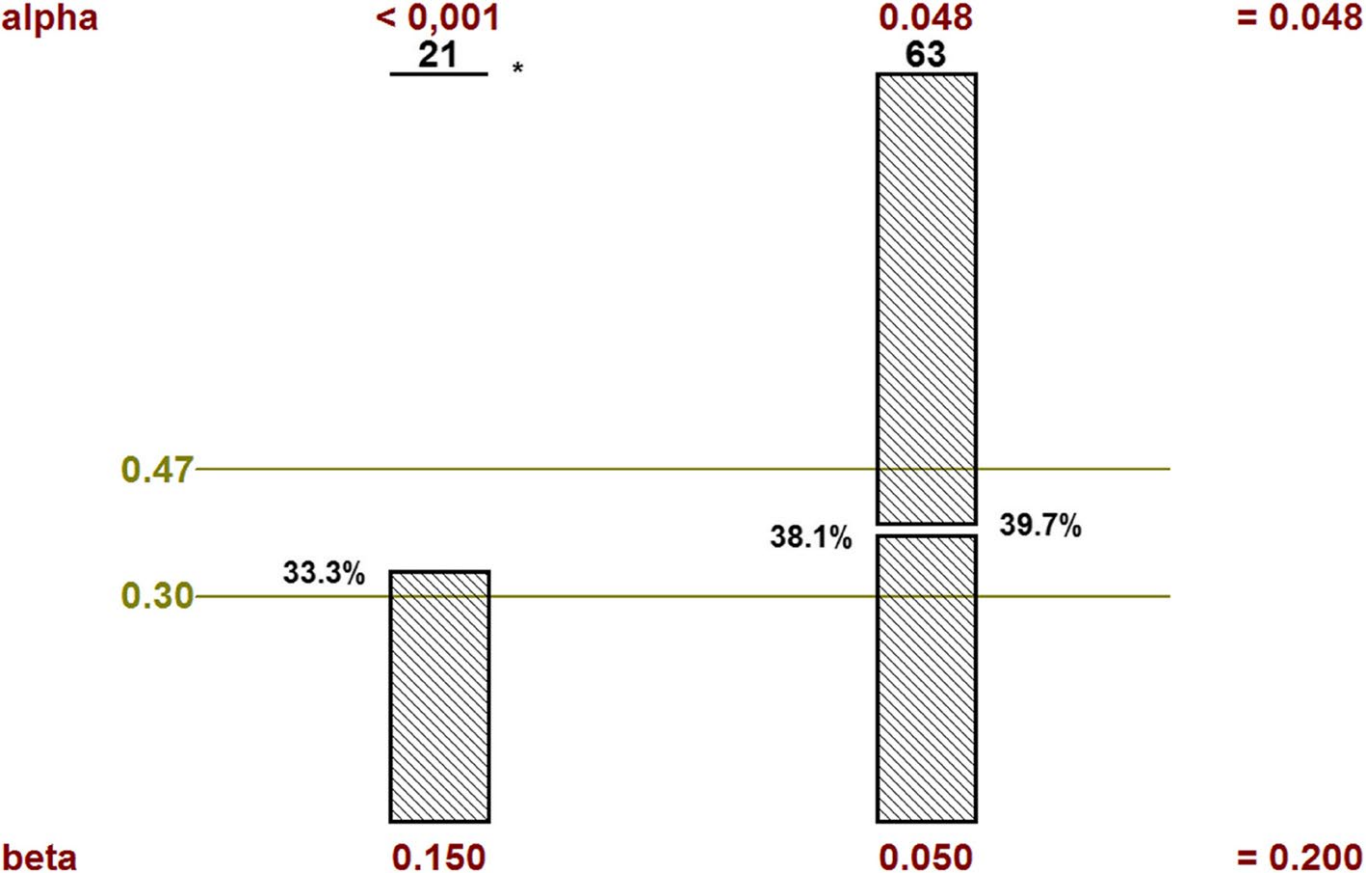
Simon two-stage Optimum design fixed in the experimental arm

According to a Simon two-stage Optimum design (with a one-sided 5% type I error and power of 80%, Fig. 3), 63 evaluable patients for objective response rate at 6 months will need to be randomized in order to test the following hypotheses:

- H0: 30% (P0) of patients with an objective response is uninteresting
- H1: 47% (P1) of patients with an objective response is interesting
- In terms of statistic the study was elaborated to test the following hypotheses in the experimental arm: H0: P ≤ P0 versus H1: P ≥ P1

Stage 1:

After randomization of the first 21 patients with a follow-up of 6 months from the date of randomization:

- if ≤ 7 (33.3%) patients are identified with an objective response, the intervention could be declared uninteresting. No more additional patient will be included and the study will be stopped.
- if ≥ 8 patients are identified with an objective response, 42 additional patients will be randomized.

Stage 2:

After recruitment of 63 evaluable patients with a follow-up of 6 months from the date of the randomization:

- if ≤ 24 (38.1%) patients are identified with an objective response, the intervention will be declared uninteresting
- if ≥ 25 (39.7%) patients are identified with an objective response, the intervention will be regarded as interesting

With an expected 10% rate of patients not evaluable at 6 months or drop out patients, it will be necessary to randomize a total of 70 patients in the experimental Arm (Arm A).

Considering the randomization ratio 2:1, it will be necessary to randomize 35 patients in the control Arm (Arm B) and then to randomized a total of 105 patients in the phase II.The control arm will serve to verify the good calibration of the P0 hypothesis made in the experimental arm and to provide true controls for translational investigations. No statistical comparison is planned between the two arms.

Randomization will be done using minimization technique (hazard compound 0.9) with stratification according to:

- Center
- Macrovascular invasion and/or extrahepatic spread: absence versus presence
- Baseline alpha-foetoprotein (AFP): < 400 versus ≥ 400 ng/mL
- ECOG-PS: 0 versus 1

At the stage 1, in parallel given that the safety profile of UCPVax and atezolizumab plus bevacizumab combination has not been evaluated in patients with HCC so far, a semi-continuous monitoring for toxicity using Pocock-type boundary will be performed (probability of early stopping: 0.05, targeted dose-limiting toxicity [DLT] rate: 0.20). Patient recruitment will be stopped and discussed with the DSMB (Data Safety Monitoring Board) after the 21 first patients included in the experimental arm (Arm A) with a minimal follow-up of 6 months.

The trial will be stopped if the number of dose limiting toxicities is equal to or exceeds bn out of n patients with completed follow-up. This boundary is equivalent to testing the null hypothesis, after each patient, that the event rate is equal to 0.2, using a one-sided level 0.019493 test [21]. After the 21 first patients included in the experimental arm (Arm A) with a minimal follow-up of 6 months, if excessive numbers (≥ 20%) of DLT are screen, that is, if the number of DLT is equal to or exceeds bn out of n patients with full follow-up, the accrual will be halted and a safety board will be convened to decide on the study continuation (Table 2). This is a Pocock-type stopping that yields the probability of crossing the boundary at most 0.05 (probability of early stopping) when the rate of DLT is equal to 0.20 (targeted acceptable DLT rate).

**Table 2:** Pocock-type stopping

Number of patients, n	1	2	3	4	5	6	7	8	9	10	11	12	13	14	15	16	17	18	19	20
Boundary, b_n_	-	-	3	4	4	4	5	5	6	6	6	6	7	7	7	8	8	8	9	9
Number of patients, n	21	22	23	24	25	26	27	28	29	30	31	32	33	34	35	36	37	38	39	40
Boundary, b_n_	9																			

An Independent Data and Safety Monitoring Committee (IDMC) will be constituted and will meet for the validation of the passage from stage 1 to stage 2; for the monitoring of toxicities and also for the reviewing of serious adverse events (SAE). DSMB will meet also in case of unexpected toxicities or in demand.

### Modality of analysis

The primary analysis will be on modified intention-to-treat (mITT) population, *i.e.* including all evaluable patients for the primary endpoint regardless of their eligibility and who have received at least one dose of treatment. Confirmative analyses will be conducted firstly in the ITT population (not assessable patients and patients with drop out between inclusion and 6 months will be considered as progressive) and secondly, in the Per Protocol population defined as patients who have received at least one dose of treatment and presenting no major deviations from the protocol.

Analyses of safety will be conducted in all patients who have received at least one dose of allocated treatment.

### Monitoring and safety

As in our previous studies [18–20], an IDMC is created in the framework of the TERTIO study. An IDMC represented by a multidisciplinary team, including physicians from relevant medical disciplines and biostatistical background, is implemented to ensure the safety and well-being of the patients exposed to study treatment through the on-going review of safety data, assure the highest integrity about operation and performance of the trial, and evaluate on-going safety data to detect the possibility of an unfavourable early treatment risk. IDMC will meet for the validation of the passage from stage 1 to stage 2 (see statistical section); for the monitoring of toxicities and also for the reviewing of SAE. IDMC will meet also in case of unexpected toxicities or in demand.

IDMC’s responsibilities are to review safety data on an on-going basis, recommend that the clinical study be stopped early if there is strong evidence that the investigational medicinal products are harming patients, and make recommendations regarding modification of the study if there is strong evidence that such change would substantially contribute to the well-being of patients. The IDMC will independently make its recommendations for continuation or termination of the trial to the appropriate Sponsor contact. The IDMC will maintain records of all its meetings and activities related to the study. These records will remain confidential until completion of the final analysis at which time they will be forwarded to the Sponsor for appropriate filing. Members will be aware of data management and quality control procedures to be confident the data are timely, accurate and complete.

## Discussion

Current clinical trials have shown that less than one patient out of three achieved clinical responses to anti–PD-L1 and anti-angiogenic in first-line treatment for patients with unresectable HCC. Combining atezolizumab plus bevacizumab with a CD4 Th1-inducer cancer vaccine derived from telomerase, in order to activate antitumor T cell immunity and bypass the immunosuppression in the tumor microenvironment in HCC, might increase the number of patients achieving a clinical benefit and the immunological efficacy.

## Data Availability

Not applicable.

## Abbreviations

DLT: Dose-limiting toxicity
ECOG-PS: Eastern Cooperative Oncology Group-performance status
eCRF: Electronic case report form
GMP: Good Manufacturing Practice
HCC: Hepatocellular carcinoma
HPV: Human papilloma virus
IDMC: Independent Data and Safety Monitoring Committee
mITT: Modified intention-to-treat
OS: Overall survival
PFS: Progression-free survival
QoL: Health-related quality of life
SAE: Serious adverse events
TERT: Telomerase reverse transcriptase
UCP: Universal Cancer Peptides

## References

[1] Calderaro J, Couchy G, Imbeaud S, Amaddeo G, Letouzé E, Blanc JF (2017). Histological subtypes of hepatocellular carcinoma are related to gene mutations and molecular tumour classification. J Hepatol.

[2] Ningarhari M, Caruso S, Hirsch TZ, Bayard Q, Franconi A, Védie AL (2021). Telomere length is key to hepatocellular carcinoma diversity and telomerase addiction is an actionable therapeutic target. J Hepatol mai.

[3] Nault JC, Mallet M, Pilati C, Calderaro J, Bioulac-Sage P, Laurent C (2013). High frequency of telomerase reverse-transcriptase promoter somatic mutations in hepatocellular carcinoma and preneoplastic lesions. Nat Commun.

[4] Finn RS, Qin S, Ikeda M, Galle PR, Ducreux M, Kim TY (2020). Atezolizumab plus Bevacizumab in Unresectable Hepatocellular Carcinoma. N Engl J Med.

[5] Cheng AL, Qin S, Ikeda M, Galle PR, Ducreux M, Kim TY (2021). Updated efficacy and safety data from IMbrave150: Atezolizumab plus bevacizumab vs. sorafenib for unresectable hepatocellular carcinoma. J Hepatol.

[6] Yau T, Kang YK, Kim TY, El-Khoueiry AB, Santoro A, Sangro B (2020). Efficacy and Safety of Nivolumab Plus Ipilimumab in Patients With Advanced Hepatocellular Carcinoma Previously Treated With Sorafenib: The CheckMate 040 Randomized Clinical Trial. JAMA Oncol.

[7] Kelley RK, Sangro B, Harris W, Ikeda M, Okusaka T, Kang YK (2021). Safety, Efficacy, and Pharmacodynamics of Tremelimumab Plus Durvalumab for Patients With Unresectable Hepatocellular Carcinoma: Randomized Expansion of a Phase I/II Study. J Clin Oncol.

[8] Abou-Alfa GK, Lau G, Kudo M, Chan SL, Kelley RK, Furuse J, et al. Tremelimumab plus Durvalumab in Unresectable Hepatocellular Carcinoma. NEJM Evidence. 2022;1(8). Available from: https://evidence.nejm.org/doi/10.1056/EVIDoa2100070. 10.1056/EVIDoa2100070 38319892

[9] Kelley RK, Rimassa L, Cheng AL, Kaseb A, Qin S, Zhu AX (2022). Cabozantinib plus atezolizumab versus sorafenib for advanced hepatocellular carcinoma (COSMIC-312): a multicentre, open-label, randomised, phase 3 trial. Lancet Oncol.

[10] Finn RS, Kudo M, Merle P, Meyer T, Qin S, Ikeda S (2022). LBA34 - Primary results from the phase III LEAP-002 study: Lenvatinib plus pembrolizumab versus lenvatinib as first-line (1L) therapy for advanced hepatocellular carcinoma (aHCC). Annal Oncol.

[11] Filderman JN, Storkus WJ (2022). Finding the right help in the tumor microenvironment. J Clin Invest.

[12] Ahrends T, Busselaar J, Severson TM, Bąbała N, de Vries E, Bovens A (2019). CD4+ T cell help creates memory CD8+ T cells with innate and help-independent recall capacities. Nat Commun.

[13] Magen A, Nie J, Ciucci T, Tamoutounour S, Zhao Y, Mehta M (2019). Single-cell profiling defines transcriptomic signatures specific to Tumor-reactive versus virus-responsive CD4+ T Cells. Cell Rep.

[14] Ferris ST, Durai V, Wu R, Theisen DJ, Ward JP, Bern MD (2020). cDC1 prime and are licensed by CD4+ T cells to induce anti-tumour immunity. Nature août.

[15] Duhen R, Fesneau O, Samson KA, Frye AK, Beymer M, Rajamanickam V (2022). PD-1 and ICOS coexpression identifies tumor-reactive CD4+ T cells in human solid tumors. J Clin Invest.

[16] Godet Y, Fabre E, Dosset M, Lamuraglia M, Levionnois E, Ravel P (2012). Analysis of spontaneous tumor-specific CD4 T-cell immunity in lung cancer using promiscuous HLA-DR telomerase-derived epitopes: potential synergistic effect with chemotherapy response. Clin Cancer Res Off J Am Assoc Cancer Res.

[17] Adotévi O, Vernerey D, Jacoulet P, Meurisse A, Laheurte C, Almotlak H (2023). Safety, Immunogenicity, and 1-Year Efficacy of Universal Cancer Peptide-Based Vaccine in Patients With Refractory Advanced Non-Small-Cell Lung Cancer: a Phase Ib/Phase IIa De-Escalation Study. J Clin Oncol Off J Am Soc Clin Oncol.

[18] Rebucci-Peixoto M, Vienot A, Adotevi O, Jacquin M, Ghiringhelli F, de la Fouchardière C (2022). A Phase II Study Evaluating the Interest to Combine UCPVax, a Telomerase CD4 TH1-Inducer Cancer Vaccine, and Atezolizumab for the Treatment of HPV Positive Cancers: VolATIL Study. Front Oncol.

[19] Kim S, Buecher B, André T, Jary M, Bidard FC, Ghiringhelli F (2020). Atezolizumab plus modified docetaxel-cisplatin-5-fluorouracil (mDCF) regimen versus mDCF in patients with metastatic or unresectable locally advanced recurrent anal squamous cell carcinoma: a randomized, non-comparative phase II SCARCE GERCOR trial. BMC Cancer.

[20] Kim S, Boustani J, Vernerey D, Vendrely V, Evesque L, Francois E (2022). Phase II INTERACT-ION study: ezabenlimab (BI 754091) and mDCF (docetaxel, cisplatin, and 5-fluorouracil) followed by chemoradiotherapy in patients with Stage III squamous cell anal carcinoma. Front Oncol.

[21] Ivanova A, Qaqish BF, Schell MJ (2005). Continuous toxicity monitoring in phase II trials in oncology. Biometrics.

